# Supplementary material for: The Burden and Risk Factors of Patellar and Achilles Tendinopathy in Youth Basketball: A Cohort Study
Source: Int J Environ Res Public Health. 2021 Sep 8;18(18):9480. doi: 10.3390/ijerph18189480 (PMC8470990; doi:10.3390/ijerph18189480)
Supplement: Supplementary file 1 [file ijerph-18-09480-s001.zip › ijerph-1353356-supplementary.pdf]

OSTRC – Patellar Tendinopathy Questionnaire

Please answer all questions regardless of whether or not you have problems with your knees. Select **(tick or circle)** the option that is most appropriate for you, and in the case that you are unsure, try to give an answer as best you can anyway.

The term "knee problems" refers to pain, ache, stiffness, swelling, instability/giving way, locking or other complaints related to one or both knees. Please note that all questions in this questionnaire refer to the previous week.

Question 1 - Have you had any difficulties participating in normal practice and game due to knee problems this past week?

a) Full participation without knee problems c) Reduced participation due to knee problems
b) Full participation but with knee problems d) Cannot participate due to knee problems

Question 2 - To what extent have you reduced your practice volume due to knee problems this past week?

a) No reduction b) To a minor extent c) To a moderate extent
d) To a major extent e) Cannot participate at all

Question 3 - To what extent have knee problems affected your performance this past week?

a) No effect b) To a minor extent c) To a moderate extent
d) To a major extent e) Cannot participate at all

Question 4 - To what extent have you experienced knee pain related to playing basketball this past week?

a) No pain b) Mild pain c) Moderate pain d) Severe pain

** If you answered "a" to all 4 questions, questionnaire is completed for the week; if otherwise please answer the following questions:*

Question 5 – Do you still experience any knee pain, especially during and/or after basketball participation?

a) Yes b) No **If "yes" please proceed to Question 6, if otherwise questionnaire is completed*

Question 6 - Is the knee pain you are reporting?

a) The same knee pain as in previous week(s) b) A return of a knee pain that had gone away
c) A knee pain that is being experienced for the first time this past week

Question 7 - On which knee do you have pain?

a) Right knee b) Left knee c) Both knees (right and left)

**Complete this section as applicable**		
	**Right knee**	**Left knee**
**Question 8**<br>**Describing the onset of your knee pain, was it:**	a) Of a gradual or sudden onset that is **unidentifiable** with any event?<br>b) Of a sudden onset that is **clearly identifiable** (e.g. impact or collision with another player)?	a) Of a gradual or sudden onset that is **unidentifiable** with any event?<br>b) Of a sudden onset that is **clearly identifiable** (e.g. impact or collision with another player)?
If your answer to Question 8 is "a" please proceed to Question 9, if otherwise questionnaire is completed		
**Question 9**<br>**Describe the location of your knee pain (you can select multiple):**	a) Front of the knee<br>b) Back of the knee<br>c) Inside of the knee (medial)<br>d) Outside of the knee (lateral)	a) Front of the knee<br>b) Back of the knee<br>c) Inside of the knee (medial)<br>d) Outside of the knee (lateral)
If your answer to Question 9 is "a" please proceed to Question 10, if otherwise questionnaire is completed		
**Question 10**<br>**Is the pain in the front of your knee on the bottom tip of your kneecap?**	a) Yes<br>b) No	a) Yes<br>b) No

OSTRC – Achilles Tendinopathy Questionnaire

Please answer all questions regardless of whether or not you have problems with your ankles. Select **(tick or circle)** the option that is most appropriate for you, and in the case that you are unsure, try to give an answer as best you can anyway.

The term "ankle problems" refers to pain, ache, stiffness, swelling, instability/giving way, locking or other complaints related to one or both ankles. Please note that all questions in this questionnaire refer to the previous week.

Question 1 - Have you had any difficulties participating in normal practice and game due to ankle problems this past week?

a) Full participation without ankle problems c) Reduced participation due to ankle problems
b) Full participation but with ankle problems d) Cannot participate due to ankle problems

Question 2 - To what extent have you reduced your practice volume due to ankle problems this past week?

a) No reduction b) To a minor extent c) To a moderate extent
d) To a major extent e) Cannot participate at all

Question 3 - To what extent have ankle problems affected your performance this past week?

a) No effect b) To a minor extent c) To a moderate extent
d) To a major extent e) Cannot participate at all

Question 4 - To what extent have you experienced ankle pain related to playing basketball this past week?

a) No pain b) Mild pain c) Moderate pain d) Severe pain

** If you answered "a" to all 4 questions, questionnaire is completed for the week; if otherwise please answer the following questions:*

Question 5 – Do you still experience any ankle pain, especially during and/or after basketball participation?

a) Yes b) No **If "yes" please proceed to Question 6, if otherwise questionnaire is completed*

Question 6 - Is the ankle pain you are reporting?

a) The same ankle pain as in previous week(s) b) A return of an ankle pain that had gone away
c) An ankle pain that is being experienced for the first time this past week

Question 7 - On which ankle do you have pain?

a) Right ankle b) Left ankle c) Both ankles (right and left)

**Complete this section as applicable**		
	**Right ankle**	**Left ankle**
**Question 8**<br>**Describing the onset of your ankle pain, was it:**	a) Of a gradual or sudden onset that is **unidentifiable** with any event?<br>b) Of a sudden onset that is **clearly identifiable** (e.g. impact or collision with another player)?	a) Of a gradual or sudden onset that is **unidentifiable** with any event?<br>b) Of a sudden onset that is **clearly identifiable** (e.g. impact or collision with another player)?
If your answer to Question 8 is "a" please proceed to Question 9, if otherwise questionnaire is completed		
**Question 9**<br>**Describe the location of your ankle pain (you can select multiple):**	a) Front of the ankle<br>b) Back of the ankle<br>c) Inside of the ankle (medial)<br>d) Outside of the ankle (lateral)	a) Front of the ankle<br>b) Back of the ankle<br>c) Inside of the ankle (medial)<br>d) Outside of the ankle (lateral)
If your answer to Question 9 is "a" please proceed to Question 10, if otherwise questionnaire is completed		
**Question 10**<br>**Is the pain in the front of your ankle on the bottom tip of your kneecap?**	a) Yes<br>b) No	a) Yes<br>b) No
